# Association between chronic lead exposure and markers of kidney injury: A systematic review and meta-analysis

**DOI:** 10.1016/j.toxrep.2024.101837

**Published:** 2024-11-29

**Authors:** Kuldip Upadhyay, Ankit Viramgami, Bhavani Shankara Bagepally, Rakesh Balachandar

**Affiliations:** a ICMR – National Institute of Occupational Health, Ahmedabad, India; b ICMR – National Institute of Epidemiology, Chennai, India

**Keywords:** Kidney injury markers, Lead exposure, N-acetyl-β-D-glucosaminidase, β-2-microglobuline, Kidney Injury Molecule-1, Systematic review

## Abstract

In view of inconsistent reports on the association between chronic lead (Pb) exposure and renal injury markers (potential site of injury), the present systematic review explored their association by reviewing studies that investigated chronic Pb-exposed and those without obvious Pb exposure. Studies reporting blood Pb levels(BLL) and biomarkers of kidney injury [i.e. N-acetyl-β-D-glucosaminidase (NAG), Micro-Globulin(μG) and others] among chronic Pb-exposed and unexposed individuals were systematically searched from digital databases available until February 26, 2024. Preferred Reporting Items of Systematic Reviews and Meta-Analysis Guidelines were adhered to during the execution. Pooled effect size and heterogeneity were estimated using the random effect model and *I2*Studies reporting blood Pb levels(BLL) and biomarkers of kidney injury [i.e. N-acetyl-β-D-glucosaminidase (NAG), Micro-Globulin(μG) and others] among chronic Pb-exposed and unexposed individuals were systematically searched from digital databases available until February 26, 2024. Preferred Reporting Items of Systematic Reviews and Meta-Analysis Guidelines were adhered to during the execution. Pooled effect size and heterogeneity were estimated using the random effect model and *I2*. Pooled quantitative analysis revealed elevated BLL [25.64 (21.59–29.70) µg/dL] Pb-exposed group. The pooled analysis confirmed significantly higher urinary NAG [0.68(0.26–1.10) units], α1μG [3.82(0.96–6.68) mg/g creatinine] β_2_μG [1.5(0.86–2.14) units and serum creatinine [0.03(0.00–0.05) mg/dL] levels in Pb-exposed group, with high heterogeneity. Current observations indicate the proximal tubular injury as the early and potential site of Pb-induced renal injury. Pb-exposed individuals experience proximal tubular injury (KIM-1, NAG) and dysfunction (β2μG, α1μG, Cystatin-C) prior to obvious clinical renal failure. Present observations should caution the policymakers towards drafting regulations for periodic screening with markers of renal injury and / or dysfunction among those chronically exposed to lead despite the certainty of evidence is very low.

## Highlights

• Chronic Pb exposure is associated with proximal tubular injury i.e. ↑ KIM-1 & NAG, earlier to onset of clinical renal failure.
• Pb exposure is associated with proximal tubular dysfunction i.e. ↑ β2μG, α1μG & Cystatin-C, prior to clinical renal failure.
• Existing literature is primarily observational and highly heterogeneous carrying high risk of bias.
• The quality of evidence from existing studies is low.

## Graphical Abstract

**Figure:**
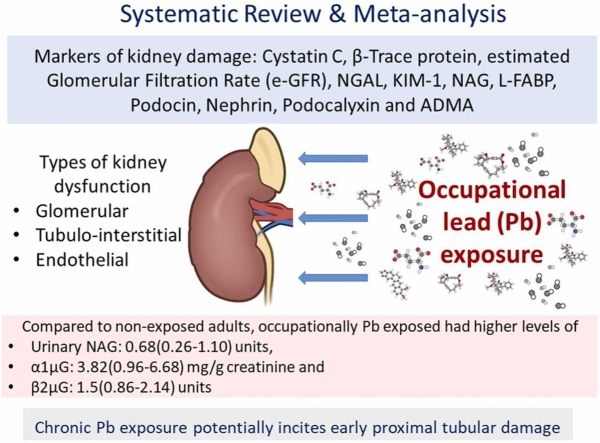

## 1 Introduction

Lead, once regarded as an industrial pollutant, in view of its profuse use, is lately recognized as an environmental pollutant [1]. Epidemiological evidence supported by a recent systematic review suggests the potentially hazardous effects of Pb exposure, particularly on reproductive systems, endocrine [2], [3], [4], [5], cardiovascular [6], immunological [7], [8], renal [9] and genotoxic [10], [11].

The burden of chronic renal dysfunction and failure has doubled since 1990, with a significant proportion of cases attributed to unknown etiology [12]. Among the suspected contributors to these cases, chronic lead (Pb) exposure has emerged as a potential etiology. The association between chronic lead exposure and kidney damage was historically observed in occupationally exposed individuals with high exposures (i.e. blood lead level > 40 µg/dL). Evidence suggests that chronic lead exposure is associated with glomerular sclerosis and interstitial fibrosis [13]. A recent systematic review supported the evidence by reporting impaired clearance of creatinine and blood urea nitrogen in adults chronically exposed to occupational Pb [14].

On a broader note, chronic renal failure can be pathophysiologically categorized based on the site of injury: tubulo-interstitial, glomerular, or endothelial dysfunction. Specific biomarkers have been identified to localize the site of injury. For tubulointerstitial damage, these include N-acetyl-β-D-glucosaminidase (NAG), Kidney Injury Molecule (KIM)-1, neutrophil gelatinase-associated lipocalin (NGAL), liver-type fatty acid-binding protein (L-FABP), or conventional markers such as glycosuria, increased urinary phosphate, and amino acids Glomerular injury can be indicated by markers like podocin, nephrin, Cystatin-C, β-Trace protein, and podocalyxin, or conventional markers such as proteinuria, reduced glomerular filtration rate (GFR), and hematuria. Endothelial dysfunction may be identified by elevated asymmetric dimethylarginine (ADMA), increased endothelin, reduced nitric oxide, and elevated intracellular adhesion molecule (sICAM) [15]. Conventional clinical markers for kidney dysfunction, viz. creatinine clearance, proteinuria, and impaired glomerular filtration, carry the disadvantage of delayed detection, lack of specificity, insensitivity to acute subtle injuries, and inability to localize the site of injury. In contrast, the novel markers mentioned above offer advantages in terms of specificity, sensitivity, and early detection of impending kidney damage.

This evidence synthesis aims to investigate the potential sites of renal injury caused by chronic Pb exposure by systematically reviewing existing literature on changes in these biomarkers among Pb-exposed individuals compared to those without obvious Pb exposure.

## 2 Methods

The study employed Preferred Reporting Items of Systematic Reviews and Meta-Analysis (PRISMA) guidelines to systematically review the existing evidence on changes in markers of kidney injury among otherwise healthy adults with evident chronic Pb exposure compared to those without evident Pb exposure [16]. The proposal is registered with PROSPERO (CRD42022354202). Biomarkers of kidney injury such as Cystatin C, β-Trace protein, estimated Glomerular Filtration Rate (e-GFR), NGAL, KIM-1, NAG, L-FABP, Podocin, Nephrin, Podocalyxin and ADMA are primarily investigated in the review. Observational studies, both cross-sectional and longitudinal, examining the relationship between chronic Pb exposure and kidney injury or dysfunction parameters were systematically searched. The search strategy utilised pre-validated terms developed following the PECOS framework, which considers Participants, Exposure (occupational Pb exposure), Controls (no evident Pb exposure), Outcomes (markers of early kidney injury/disease), and study design. Digital repositories such as Embase, Scopus, and PubMed were queried to optimise precision and sensitivity. Detailed search strategies and terms are included in Tables S1–S3. The initial search was conducted in August 2022 and updated on February 26, 2024. A lateral search of included study bibliographies identified additional relevant studies. Exclusion criteria encompassed preclinical studies (animal/cell line), studies involving acute lead poisoning, participants with congenital or acquired kidney disorders, endocrine disturbances, and non-original research (reviews, commentaries, letters, and editorials). This comprehensive review ensures a focused evaluation of chronic Pb exposure and its implications for kidney health.

### 2.1 Screening and reviewing of studies

All digital records were pooled and screened independently by three reviewers (AV, KU & RB) using the cloud-based “Rayyan intelligent systematic review” [17]. Duplicate records were manually excluded with the assistance of Rayyan’s deduplication feature. Full-text articles that met the predefined inclusion criteria were reviewed independently, and relevant data were meticulously extracted. Any conflicts or discrepancies arising during the review process were resolved through mutual consensus among the reviewers.

### 2.2 Data collection, extraction, analysis and management

A structured data extraction sheet (developed using Google Sheets) was used to record participant details, occupational exposure, and outcome parameters, including measures of central tendency and variability for each included study. To ensure consistency, standard conversion factors were applied to transform outcome parameters reported in non-standard units into standardised units, such as mg/dL, μg/dL, µg/g creatinine, and pg/dL, wherever applicable. Studies reporting outcomes in similar units were grouped together to calculate pooled estimates. The findings were uniformly documented as mean values with standard deviations through conventional transformations [18]. For studies reporting multiple exposed groups, a grand mean was calculated and used for the quantitative analysis to maintain consistency [19], [20], [21]. The pooled mean difference, along with its corresponding 95 % confidence interval, between the chronic Pb-exposed group and the control group was calculated using the generic inverse variance method. The meta-analysis employed a random-effects model to account for the high heterogeneity anticipated among the studies. This approach ensured a robust and comprehensive analysis of the data.

### 2.3 Heterogeneity, meta-regression, sensitivity and subgroup analysis

The evaluation of heterogeneity among studies, along with subgroup analysis, sensitivity analysis, and meta-regression analysis, was conducted following established procedures. Subgroup analyses were performed when at least two primary studies were available within a specific subgroup, and meta-regression analyses were undertaken for variables such as the percentage of male participants, duration of Pb exposure, and mean age of the exposed group when at least ten studies were available [22]. These analyses aimed to assess the impact of these variables on heterogeneity. Sensitivity analyses included exploring the effects of simultaneous exposure to other heavy metals and the country of reporting on the parameters of interest. Subgroup analyses noted changes in mean differences and heterogeneity relative to the overall pooled estimate to identify subgroup-specific influences. A leave-one-out meta-analysis was conducted to evaluate the influence of individual studies on the overall pooled effect by iteratively excluding one study at a time [22]. Heterogeneity was assessed using the *I*² statistic, where values greater than 25 % or a Cochrane-Q p-value < 0.1 indicated significant heterogeneity among studies. Funnel plots and contour-enhanced funnel plots were employed to assess publication bias and explore potential sources of heterogeneity when at least ten studies were available [23].

All statistical analyses were performed using Stata version 17 [24]. A p-value < 0.05 was considered statistically significant, except for subgroup analyses and heterogeneity tests, where a p-value < 0.10 was deemed significant. This rigorous approach ensured robust evaluation of variability and bias across included studies.

### 2.4 Assessment of the risk of bias in included studies

The risk of bias in the included studies was independently evaluated by the authors using the Newcastle-Ottawa Scale (NOS) [25]. To ensure consistency and rigour in the assessment, a Standard Operating Procedure (SOP) specific to this review was developed, adhering to the guidelines outlined in the NOS (details provided in the supplementary material). The NOS was employed to systematically assess the risk of bias across three domains: participant selection, comparability of cases and controls, and exposure assessment. Each study was rated based on defined criteria, including the clarity and appropriateness of case and control definitions, their representativeness, comparability, and the objectivity of exposure measurement. This structured approach ensured a standardised and transparent evaluation of the methodological quality of the included studies.

### 2.5 Certainty of evidence assessment

The level of certainty for each outcome parameter included in this systematic review was assessed using the Grading of Recommendations Assessment, Development, and Evaluation (GRADE) guidelines [26]. This tool provides a structured approach to evaluate the quality of evidence, facilitating a clearer interpretation of the results. GRADE assessment categorises the certainty of evidence into four levels: High, Moderate, Low, and Very Low, based on factors such as risk of bias, publication bias, imprecision, and inconsistency in the findings. Observational studies typically start with a Low or Very Low rating, which may be downgraded further or upgraded depending on the overall quality, robustness, and magnitude of the evidence. The GRADE framework also helps summarize the findings in a consistent and transparent manner, enhancing the interpretability and applicability of the results. [26].

## 3 Results

**Fig. 1:**
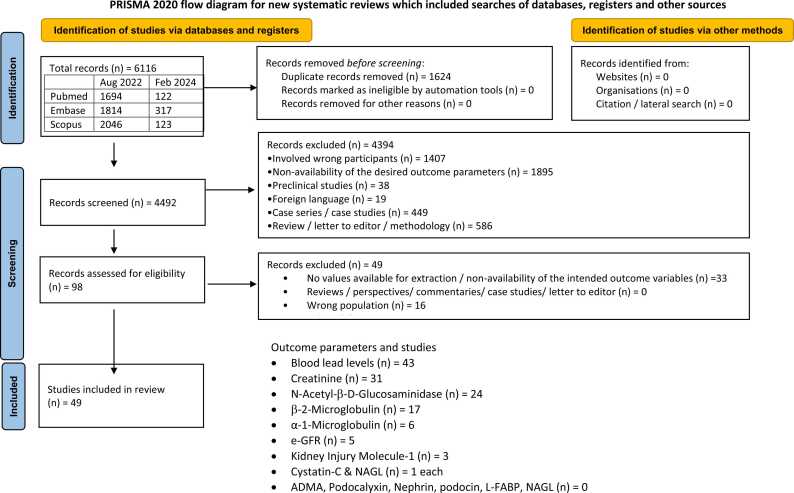
PRISMA 2020 [16] flow chart. Flow chart illustrating the number of citations / articles included and excluded at various stages.

The electronic search initially identified 6116 citations. After removing duplicates and screening titles and abstracts, ninety-eight studies were selected for full-text review. Of these, forty-nine studies met the inclusion and exclusion criteria and were included in the systematic review. The PRISMA flowchart (Fig. 1) provides a detailed overview of the study selection process, including the number of studies excluded at each stage and the reasons for their exclusion.

### 3.1 Description of studies

The details of the included studies, such as sample size, demographics, occupational exposure, and outcome indicators of interest, are summarized in Table 1. Most studies reported that the mean values of the outcome variables for both the exposed and control groups fell within the recommended normal ranges, suggesting an absence of obvious clinical renal failure or disease among participants. (i.e. creatinine 0.6–1.2 mg/dL for males and 0.5–1.1 mg/dL for females; α-1-Microglobulin <35 mg/g creatinine [27]; β-2-Microglobulin ≤300 mcg/L [28] / < 300 mcg/g crt [29] / ≤132 mcg/g creat [30]; KIM 1 < 1 ng/mL urine & may increase more than 3 ng/mL during acute kidney injury [31], urinary NAG: 3.3–4.1 U/L; 1.0–4.6 U/g creatinine [32]). Notably, none of the studies investigated ADMA, Podocalyxin, Nephrin, Podocin, or L-FABP as biomarkers. Chronic Pb exposure sources were diverse, spanning lead battery and allied industries, mining, heavy metal industrial processes, electronics and home appliance manufacturing, recycling industries, automobile, jewellery, steel plants, glasswork, and paint industries. The reported mean duration of exposure ranged from 1.85 to 25.50 years. Some studies also confirmed simultaneous exposure to other heavy metals such as arsenic (As), copper (Cu), chromium (Cr), and cadmium (Cd) among chronically Pb-exposed workers. The geographic availability of studies was limited to regions in Latin America (Brazil), North America (Canada, southeastern USA), Asia (India, Iran, China, Japan, South Korea, Singapore), and Europe (Turkey, Sweden, Belgium, Poland, Germany, Czechoslovakia, Italy, Netherlands). However, there were no studies from Australia and limited representation from Africa and significant parts of Asia.

**Table 1:** Description of the original studies included in the present systematic review.

Citation (study site / country)	Outcome parameters reported	Sample size (n) & % of male participants	Mean (SD) duration of employment among the exposed in years, and simultaneous exposure to other heavy metals	Mean (SD) age of the participants in years	Type of industry, the exposed group were employed
[33] (Egypt)	Pb* , Creatinine, KIM−1 *	Exposed: n = 35; 100 % male Control: n = 35; 100 % male	Mean (SD) duration of exposure is 12.69 (6.27) years	Mean (SD) age of exposed group is 41.31 (6.38) years; Mean (SD) age of exposed group is 41.6 (6.52) years	Electronics and Home appliances manufacturing company
[34] (India)	Pb* , Creatinine*	Exposed: n = 42 Control: n = 50			Spray Painting
[35] (Nigeria)	Pb, Creatinine	Exposed: n = 89; 56.1 % male Control: n = 65; 53.8 % male	Exposed additionally to Hg, Cd, Cr, As	Age range of exposed group is 10–60 years; Age range of control group is 10–60 years	Mining
[36] (India)	Pb* , Creatinine*	Exposed: n = 42 Control: n = 50			Silver Jewelry
[37] (Nigeria)^	Pb, Creatinine, NAG	Exposed: n = 100; Control: n = 50;	Range of exposure is 3–7 years	Age range of exposed group is 23–43 years; age range of control group is 20–50 years	Lead smelting and battery manufacturing plant
[38] (USA)	Pb, Creatinine, e-GFR	Exposed: n = 52 Control: n = 726	Mean (SD) duration of exposure is 14.1(12.2) years	Mean (SD) age of exposed group is 55.8 (10.5) years; Mean (SD) age of control group is 45.8 (14.4) years	Lead smelting
[39] (China)	Pb* , Creatinine, Cystatin-C* , α−1-Microglobulin* , β−2-Microglobulin* , e-GFR, KIM−1 * , NAG*	Exposed: n = 92; 54.3 % male Control: n = 92; 47.8 % male		Mean (SD) age of exposed group is 21.2 (2.86) years; Mean (SD) age of control group is 20.85 (2.61) years	Light-bulb factory
[40] (Turkey)^	Pb* , α−1-Microglobulin, β−2-Microglobulin, NAG	Exposed: n = 53; Control: n = 40;	Mean (SD) duration of exposure is 15.8 (7.3) years; Exposed additionally to Cd	Mean (SD) age of exposed group is 45.5 (16.8) years; Mean (SD) age of control group is 45.3 (17.5) years	Steel plant (foundry man and crane operators)
[20] (Poland)^	Pb, β−2-Microglobulin	Exposed: n = 35; Control: n = 16;	Exposed additionally to As	Mean (SD) age of exposed group is 44.3 (10.3) years; Mean (SD) age of control group is 46.1 (9.6) years	Copper smelter plant (Furnace, smelting, electrolysis)
[13] (India)	Pb* , Creatinine*	Exposed: n = 53; Control: n = 42		Mean (SD) age of exposed group is 30.9 (7.7) years; Mean (SD) age of control group is 30.1 (1.2) years	Automobile mechanics, Battery repairing, Petrol station workers
[41] (China)^$^	Pb, NAG	Exposed: 319 male & 142 female Control: 126 male and 49 female	Mean duration of exposure among men is 22.26 years Mean duration of exposure among women is 18.05 years	Exposed group: Mean (SD) age of men is 35.58 (7.14) years & women is 41.31(8.02) years; Control group: Mean (SD) age of men is 34(10.6) years and women is 41.6(9.39) years	Battery storage plants
[42] (China)	Pb* , Creatinine* , e-GFR*	Exposed: n = 190; Control: n = 80			welder, painter, battery workers, radiator repairers, petrol workers
[43] (China)	Pb* , NAG*	Exposed: n = 155; Control: n = 36	Mean (SD) duration of exposure is 18.07 (9.39) years	Mean (SD) age of exposed group is 43.5 (8.6) years;Mean (SD) age of control group is 45 (9.8) years	Automobile battery storage plant
[44] (China)	Pb* , β−2-Microglobulin* , NAG*	Exposed: n = 135 Control: n = 143	Mean (SD) duration of exposure is 5.8 (4.4) years	Mean (SD) age of exposed group is 28.7 (6.6) years;Mean (SD) age of control group is 27 (8.5) years	Battery storage plant
[45] (India)	Pb* , Creatinine	Exposed: n = 90 Control: n = 35		Mean (SD) age of exposed group is 39.0 (8.5) years	Battery manufacturing, Silver jewelry, Spray painters
[46] (Nigeria)	Pb* , Creatinine*	Exposed: n = 25 Control: n = 25			Artisan
[47] (Egypt)	Pb, α−1-Microglobulin, NAG	Exposed: n = 81 Control: n = 75			Electric cable manufacturing
[48] (Turkey)	Pb* , Creatinine	Exposed: n = 79 Control: n = 71	Mean (SD) duration of exposure is 3.4 (1.7) years	Mean (SD) age of exposed group is 17.3 (1.0) years;Mean (SD) age of control group is 17.0 (1.1)	Auto repair workshop
[49] (South Korea)	Pb, Creatinine, NAG	Exposed: n = 803; 79.6 % male Control: n = 135; 91.9 % male	Mean (SD) duration of exposure is 8.2 (6.5) years; Exposed additionally to Cd	Mean (SD) age of exposed group is 40.4 (10.1) years;Mean (SD) age of control group is 34.5 (9.1) years	Lead battery, lead oxide, lead crystal, radiator manufacture, and secondary lead smelting
[50] (Egypt)	Pb* , Creatinine, β−2-Microglobulin, NAG*	Exposed: n = 43 Control: n = 52	Mean (SD) duration of exposure is 10.4 (3.7) years	Mean (SD) age of exposed group is 33 (5.6) years;Mean (SD) age of control group is 31 (4.2) years	Automobile exhaust exposure
[51] (Sweden)	Pb, β−2-Microglobulin, NAG	Exposed: n = 22; 100 % male Control: n = 11; 100 % male	Mean (SD) duration of exposure is 25.5 (5.63) years; Exposed additionally to Cd		Smelting plant
[52] (South Korea)	Pb* , Creatinine, α−1-Microglobulin* , β−2-Microglobulin, NAG	Exposed: n = 56 Control: n = 64	Mean (SD) duration of exposure is 8.3 (4.6) years	Mean (SD) age of exposed group is 42.9 (7.9) years;Mean (SD) age of control group is 44.2 (8.6) years	Lead smelting
[53] (Belgium)	Pb, β−2-Microglobulin	Exposed: n = 47 Control: n = 55	Mean (SD) duration of exposure is 15.9 (7.5) years; Exposed additionally to Cd	Mean (SD) age of exposed group is 42.3 (6.6) years;Mean (SD) age of control group is 43 (7.92) years	Lead smelting
[54] (India)	Pb, Creatinine, β−2-Microglobulin, NAG Pb, α−1-Microglobulin, β−2-Microglobulin	Exposed: n = 22; 100 % male Control: n = 27; 100 % male		Mean (SD) age of exposed group is 31.7 (6.45) years;Mean (SD) age of control group is 32.5 (7.96) years	Automobile mechanics
[55] (Singapore)	Pb, Creatinine, β−2-Microglobulin, NAG Pb, α−1-Microglobulin, β−2-Microglobulin	Exposed: n = 128 Control: n = 93	Mean (SD) duration of exposure is 1.85 (0.47) years; Exposed additionally to Cd	Mean (SD) age of exposed group is 28.5 (7.27) years;Mean (SD) age of control group is 25.7 (6.37) years	Lead stabilizer manufacturing
[56] (Brazil)	Pb, Creatinine, NAG*	Exposed: n = 166 Control: n = 60	Mean (SD) duration of exposure is 4.5 (26.29) years	Mean age of exposed group is 33.4 years;Mean (SD) age of control group is 33.5 years	Lead battery manufacturing and recycling
[57] (Belgium)	Pb* , Creatinine, β−2-Microglobulin, NAG*	Exposed: n = 76; 100 % male Control: n = 68; 100 % male	Mean (SD) duration of exposure is 17.7 (7.3) years; Exposed additionally to Cd	Mean (SD) age of exposed group is 43.6 (7.8) years;Mean (SD) age of control group is 43.4 [9] years	Lead smelting
[58] (Singapore)	Pb, NAG	Exposed: n = 128 Control: n = 152	Mean (SD) duration of exposure is 2.9 (3.39) years	Mean (SD) age of exposed group is 28.4 (7.28) years;Mean (SD) age of control group is 27.1 (7.1) years	Lead stabilizer manufacturing
[59] (Germany)	Pb* , Creatinine, α−1-Microglobulin, β−2-Microglobulin, NAG	Exposed: n = 82; 100 % male Control: n = 44; 100 % male	Mean (SD) duration of exposure is 7 [6] years	Mean (SD) age of exposed group is 30 (7.33) years;Mean (SD) age of control group is 29 (13.5) years	Pb dust accumulator plant
[60] (Japan)	Pb, Creatinine, α−1-Microglobulin	Exposed: n = 83 Control: n = 16	Mean (SD) duration of exposure is 10.14 (11.73) years	Mean (SD) age of exposed group is 45.25 (13.48) years;Mean (SD) age of control group is 40.5 [11] years	Pb solder and secondary Pb refinery
[61] (Sweden)	Pb, β−2-Microglobulin, NAG	Exposed: n = 100 Control: n = 41	Mean (SD) duration of exposure is 19.79 (12.17) years	Mean (SD) age of exposed group is 46.55 (17.73) years;Mean (SD) age of control group is 49.61 (16.12) years	Pb smelter
[62] (Belgium)	Pb, Creatinine, β−2-Microglobulin, NAG	Exposed: n = 98 Control: n = 85	Mean (SD) duration of exposure is 10.6 (8.1) years; Exposed additionally to Cd	Mean (SD) age of exposed group is 37.7 (8.3) years;Mean (SD) age of control group is 38.8 (8.7) years	Pb acid battery
[63] (Czechoslovakia)	Pb, β−2-Microglobulin	Exposed: n = 45 Control: n = 23	Mean (SD) duration of exposure is 14 [7] years	Mean (SD) age of exposed group is 39 [9] years;Mean (SD) age of control group is 42 [10] years	Glass work
[64] (Japan)	Pb, Creatinine, NAG	Exposed: n = 26; 100 % male Control: n = 20; 100 % male			Pb refinery
[65] (China)^a^	Pb, β−2-Microglobulin*	Exposed: n = 40; 90 % male Control: population mean	Mean (SD) duration of exposure is 5.4 (2.35) years		Pb smelter
[66] (Italy)	Pb, NAG*	Exposed: n = 20 Control: n = 20	Mean (SD) duration of exposure is 9.6 (4.1) years		Pb battery
[67] (Brazil)	Pb* , Creatinine* ,	Exposed: n = 52 Control: n = 44		Mean (SD) age of exposed group is 44.9 (9.5) years; Mean (SD) age of control group is 43.4 (8.9) years	Lead smelting
[68] (Netherland)	Pb* , Creatinine, β−2-Microglobulin^#^, NAG	Exposed: n = 155 Control: n = 126	Exposed additionally to Cd		
[69] (USA)	Pb, NAG	Exposed: n = 29 Control: n = 68			Lead paint,
[70] (UAE)	Pb* , Creatinine,	Exposed: n = 100 Control: n = 100	Mean (SD) duration of exposure is 8.3 (5.9) years	Mean (SD) age of exposed group is 34.6 [8] years; Mean (SD) age of control group is 35.5 (9.4) years	Participants were Taxi drivers, fuel gas filling workers, garage workers, workers in chemical printing, building & metal industries
[71] (Europe)	Pb* , Creatinine	Exposed: n = 100 Control: n = 95		Mean (SD) age of exposed group is 38.35 (11.64) years; Mean (SD) age of control group is 37.65 (16.9) years	Mechanical workshop workers
[72] (Pakistan)	Pb* , Creatinine	Exposed: n = 87 Control: n = 61	Exposed for 3 – 24 years	Mean (SD) age of exposed group is 40 [10] years; Mean (SD) age of control group is 38 [11] years	Lead smelting workers
[73] (Nigeria)	Pb* , Creatinine	Exposed: n = 25 Control: n = 25	Simultaneously exposed o Cadmium and Nickel at workplace		Paint factory artisans
[74] (China)	β−2-Microglobulin* & NAG	Exposed: n = 290 Control: n = 91	Mean (range) duration of exposure is 1.6 years (1 month ∼ 7 years)	Mean (range) age of exposed group is 36 [20], [21], [22], [23], [24], [25], [26], [27], [28], [29], [30], [31], [32], [33], [34], [35], [36], [37], [38], [40], [42], [43], [44], [45], [46], [48], [49], [50], [51], [52], [53], [54], [55], [56], [57], [58], [59], [60], [61], [62], [63], [64], [66], [67], [68] years; Mean (SD) age of control group is 33 [22], [23], [24], [25], [26], [27], [28], [29], [30], [31], [32], [33], [34], [36], [37], [38], [42], [43], [44], [45], [46], [48], [50], [51], [52], [53], [54], [55], [56], [57], [58], [59], [60], [61], [62], [64], [66], [67], [68] years	Battery storage plant
[75] (Turkey)	Urinary Pb* , Creatinine	Exposed: n = 15 Control: n = 15		Mean (SD) age of exposed group is 32 (6.1) years; Mean (SD) age of control group is 21.5 (1.1) years	Gasoline station workers
[21] (India)^	Pb* , Creatinine	Exposed: n = 122 Control: n = 122	Engaged in painting activity for more than ≥ 5 years	Aged between 16 – 65 years	Painters
[76] (Iran)	Pb* , Creatinine	Exposed: n = 78, male Control: n = 78, male	Mean (SD) duration of exposure is 4.05 (2.13) years	Mean (SD) age of exposed group is 34.92 (7.08) years; Mean (SD) age of control group is 33.82 (5.47) years	Battery
[77] (Nigeria)	NAGL	Exposed: n = 162, male Control: n = 81, male		Mean (SD) age of exposed group is 47.27 (9.99) years; Mean (SD) age of control group is 48.94 (10.34) years	Automobile mechanics
[10] (Egypt)	Urinary Pb. KIM−1 *	Exposed: n = 147, male Control: n = 30, male	Mean (SD) duration of exposure is 21.3 (10.6) years	Mean (SD) age of exposed group is 47.2 (9.5) years; Mean (SD) age of control group is 47.2 (9.5) years	Automobile mechanics

*studies reporting significant difference between the occupationally Pb-exposed group and control group

$ Zheng et al. reported exposed and unexposed (control) separately for male and female participants

# Vershoor et al. [68] reported serum β-2-Microglobulin, while the other studies reported urinary β-2-Microglobulin levels

^ details of sex not available in the study

a Study compared exposed against population mean

### 3.2 Risk of bias assessment

The assessment of bias related to participant selection, exposure assessment, and outcome parameters was conducted using the Newcastle-Ottawa Scale (NOS) framework (Table 2). A significant risk of bias in participant selection was observed across most studies, primarily due to inadequate documentation regarding the representativeness of both exposed and control participants. Specifically, studies often lacked clear confirmation of exposure status among the exposed group and details on prior Pb exposure or history of renal dysfunction in the control group.

**Table 2:** Newcastle Ottawa scale for assessing the risk of bias in the included studies.

Study	Case definition	Case representativeness	Control selection	Control definition	Group comparability	Exposure ascertainment	Ascertainment method similarities	Non- Response rate	Total ^
[33]	-	-	-	-	*	-	*	-	2
[34]	-	-	-	-	*	-	*	-	2
[35]	-	-	*	-	*	-	*	-	3
[36]	-	-	-	-	*	-	*	-	2
[37]	-	-	*	-	*	-	*	-	3
[38]	-	-	*	-	*	-	*	-	3
[39]	*	*	*	-	*	-	*	-	5
[40]	-	*	-	-	*	-	*	-	3
[20]	-	-	-	-	*	-	*	-	2
[13]	-	-	-	-	*	-	*	-	2
[41]	-	-	-	-	*	-	*	-	2
[42]	-	*	-	-	*	-	*	-	3
[43]	*	*	-	-	*	*	*	-	5
[44]	-	-	*	-	*	-	*	-	3
[45]	-	-	-	-	*	-	*	-	2
[46]	-	-	-	-	*	-	*	-	2
[47]	-	-	-	-	*	-	*	-	2
[48]	-	-	-	-	*	-	*	-	2
[49]	-	-	*	-	*	-	*	-	3
[50]	-	-	-	-	*	-	*	-	2
[51]	-	-	-	-	*	-	*	-	2
[52]	-	-	-	-	*	*	*	-	3
[53]	-	-	-	-	*	-	*	-	2
[54]	-	-	*	-	*	-	*	-	3
[55]	*	*	*	-	*	*	*	-	6
[56]	-	-	-	-	*	-	*	-	2
[57]	-	-	-	-	*	-	*	-	2
[58]	*	-	-	-	*	-	*	-	3
[59]	-	-	*	-	*	-	*	-	3
[60]	*	*	-	-	*	-	*	-	4
[61]	-	-	*	-	*	-	*	-	3
[62]	-	-	-	-	*	-	*	-	2
[63]	*	*	-	-	*	-	*	-	4
[64]	*	*	-	-	*	-	*	-	4
[65]	-	-	*	-	*	-	*	-	3
[66]	-	-	-	-	*	-	*	-	2
[67]	-	*	-	-	*	-	*	-	3
[68]	-	-	-	-	*	-	*	-	2
[69]	-	-	*	-	*	-	*	-	3
[70]	-	*	-	-	-	-	*	-	2
[71]	-	-	*	-	*	-	*	-	3
[72]	-	*	-	-	-	-	*	-	2
[73]	*	*	-	-	*	-	*	-	4
[74]	-	*	-	-	-	-	*	-	2
[75]	*	*	-	-	*	-	*	-	4
[21]	-	-	-	-	-	-	*	-	1
[76]	*	*	*	*	-	-	*	-	5
[77]	-	*	-	-	-	-	*	-	2
[10]	*	*	*	-	-	*	*	-	5
Percent of studies with risk of bias	22.45	34.69	30.61	2.04	85.71	8.16	100.00	22.45	

^ Total number of stars obtained by each study

Moreover, while all studies employed consistent methodologies to compare blood Pb levels and renal function parameters between the exposed and control groups, biases in participant selection were prevalent. These biases were characterized by the use of convenience sampling, incomplete descriptions of control participants, and failure to specify recruitment sources for controls [56], [60], [68]. Insufficient documentation regarding exposure records and representativeness further compounded these issues [56], [60], [68]. Additionally, all included studies exhibited a high risk of bias in exposure assessment due to the non-blinded status of interviewers, potentially influencing data collection and interpretation. Collectively, these factors resulted in an overall high risk of bias among the included studies.

### 3.3 Certainty of evidence

The certainty of evidence for the relationship between chronic lead (Pb) exposure and biomarkers of kidney injury is rated as very low according to the GRADE assessment. A detailed evidence profile and summary of findings are presented in Table S3. The very low certainty of evidence across most outcome indicators stems from serious risks of bias, publication bias, and other potential sources of bias, as identified through NOS profiling, funnel plots, and contour-enhanced funnel plots, respectively Additionally, the certainty of evidence is further downgraded due to narrow estimates of mean differences combined with wide 95 % confidence intervals, reflecting inconsistencies and imprecision in the reported results. Collectively, these factors underscore the need for robust, high-quality studies to establish stronger evidence for the observed associations.

## 4 Results of individual outcome parameters

### 4.1 BLL

**Fig. 2:**
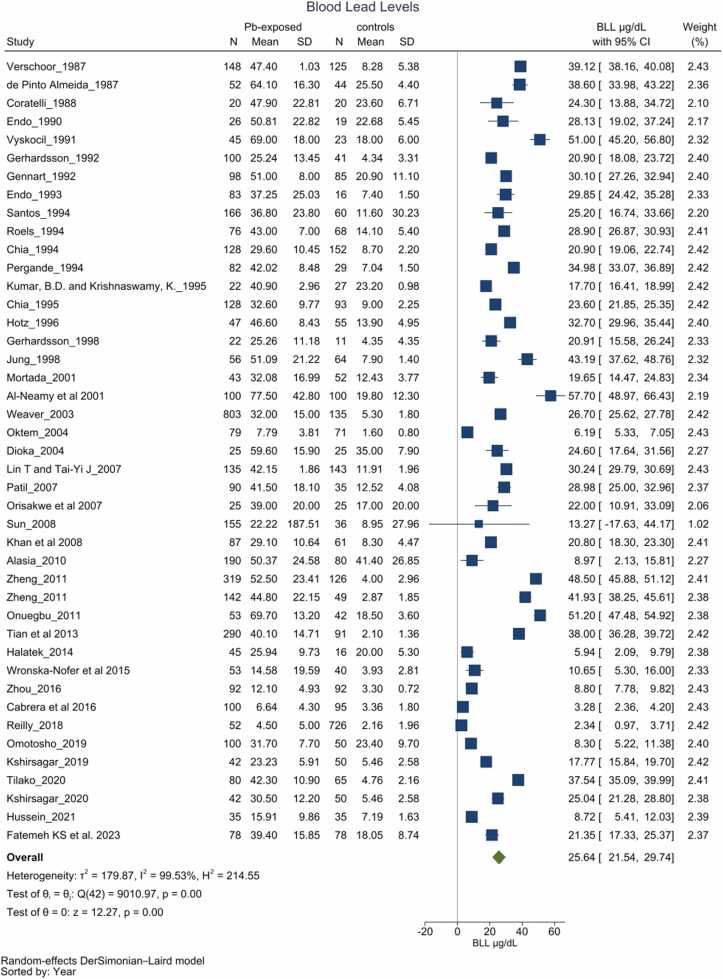
Forest plot for blood lead levels. Group differences in blood lead levels (BLL) between Pb-exposed and unexposed group. Forest plot demonstrates significantly higher BLL (25.64 µg/Dl with 95 % CI, 21.54–29.74 among Pb-exposed individuals, However the heterogeneity among the studies were unacceptably high (*I*^2^ = 99.53 %).

Forty-three [61] studies reported changes in BLL among individuals with chronic Pb exposure compared to their respective comparator groups. [13], [20], [33], [34], [35], [36], [37], [38], [39], [40], [41], [42], [43], [44], [45], [46], [48], [49], [50], [51], [52], [53], [54], [55], [56], [57], [58], [59], [60], [61], [62], [63], [64], [66], [67], [68], [70], [71], [72], [73], [74], [75], [76]. The chronic Pb-exposed group demonstrated significantly higher BLL compared to the respective control group across all included studies (p < 0.05), consistent with the pooled quantitative analysis. The mean BLL for the chronic Pb-exposed group was 25.64 µg/dL (21.54–29.74, *I*^2^=99.53 %) higher than those that without obvious Pb exposure. (Fig. 2). Subgroup analyses, which accounted for a history of simultaneous exposure to other heavy metals and the country of reporting, yielded results consistent with the overall pooled findings, indicating that these variables alone could not fully account for the observed heterogeneity across studies (Fig. S1). Furthermore, meta-regression analysis incorporating participant age did not substantially diminish heterogeneity or alter the outcomes. The asymmetry in funnel plot suggests potential publication bias (*p* = 0.766), alongside other biases indicated by the contour-enhanced funnel plot (Fig. S2).

### 4.2 Serum creatinine

**Fig. 3:**
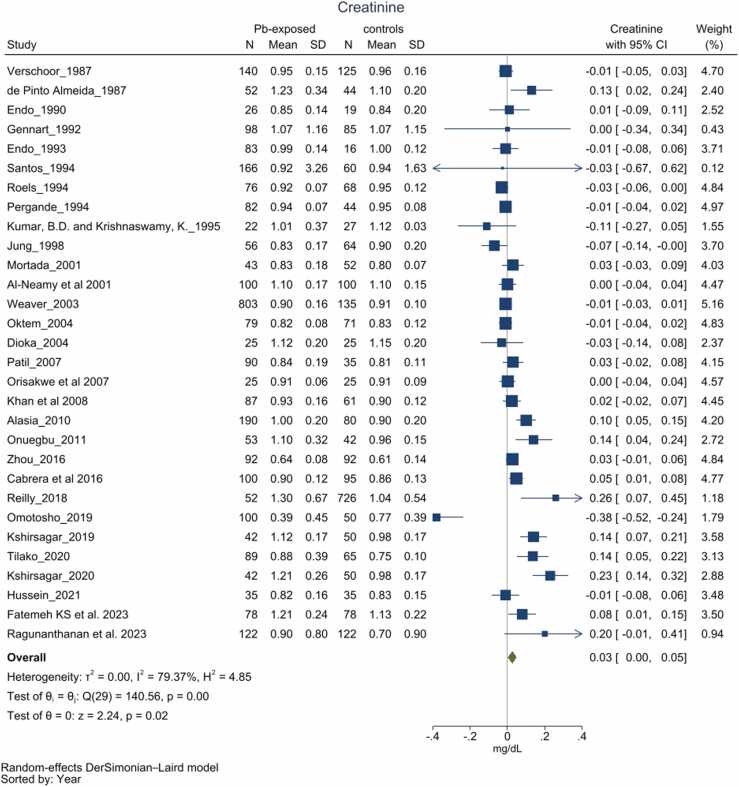
Forest plot for serum creatinine. Group differences in serum creatinine between Pb-exposed and unexposed group. Forest plot demonstrates trend of higher creatinine (0.03 mg/dL with 95 % CI 0.00–0.05) among Pb-exposed group, However the heterogeneity among the studies were unacceptably highly (*I*^2^ = 79.37 %).

Thirty-one [32] studies reported differences in creatinine levels among chronic Pb-exposed as compared to their respective comparator groups [13], [21], [33], [34], [35], [36], [37], [38], [39], [42], [45], [46], [48], [49], [50], [52], [54], [56], [57], [59], [60], [62], [64], [67], [68], [70], [71], [72], [73], [75], [76]. Primary studies observed mixed differences between the groups, with some differences being statistically not significant (p > 0.05). The pooled analysis revealed that serum creatinine levels were 0.03 mg/dL (<0.00–0.05, *I*^2^=79.37 %) higher among the individuals with chronic Pb exposure compared to the control group (Fig. 3). Subgroup analyses, accounting for simultaneous exposure to other heavy metals and the country of reporting, yielded results consistent with the pooled estimates, indicating these variables failed to entirely explain the observed heterogeneity across studies (Fig. S3). Leave-Out-One Meta-Analysis (LOOMA) demonstrated that excluding certain individual studies significantly impacted the overall results, indicating potential inconsistency among the findings of included studies (Fig. S4). Meta-regression analysis, which included participant age as a covariate, did not substantially diminish heterogeneity or alter the outcomes. Furthermore, the asymmetric funnel plot suggests potential publication bias (*p* = 0.2), alongside other biases indicated by the contour-enhanced funnel plot (Fig. S5).

### 4.3 N-Acetyl-beta-D-Glucosaminidase (NAG)

**Fig. 4:**
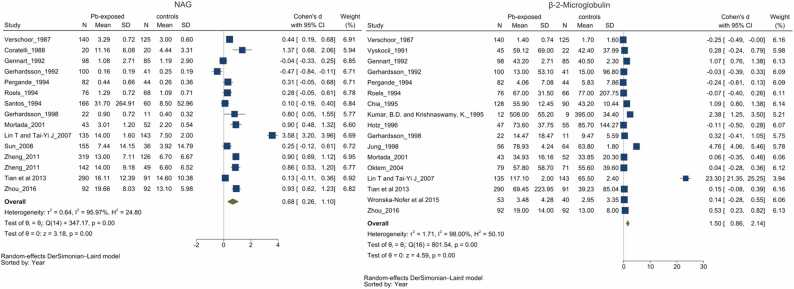
Forest plot for NAG & β-2-Microglobulin reported as standardized mean differences. Group differences in NAG (Fig. 4a) and β-2-Microglobulin (Fig. 4b) between Pb-exposed and unexposed group. Forest plot demonstrates significantly higher urinary NAG (0.68 with 95 % CI 0.26–1.10) and β-2-microglobulin (1.5 with 95 % CI 0.86–2.14) levels among Pb-exposed group, However the heterogeneity among the studies were unacceptably highly (*I*^2^ > 95 %).

Twenty-four [25] reported differences in urinary NAG levels among chronic Pb-exposed as compared to their respective comparator groups [37], [39], [40], [41], [43], [44], [47], [48], [49], [50], [51], [52], [54], [56], [57], [58], [59], [61], [62], [64], [66], [68], [69], [74]. All but two studies [62], [61] observed higher NAG levels among Pb-exposed individuals compared to controls, with few studies reporting statistically significant differences (p < 0.05), consistent with the pooled estimate. In view of the heterogeneous reporting of outcome parameters (i.e. U/g creatinine, mmol/hr/mmol of creatinine, U/mmol creatinine and U/L), the standardized mean difference was calculated to estimate the pooled effect. Meta-analysis of 15 studies revealed pooled SMD of 0.68 (0.26–1.10, *I*^2^=95.97 %) higher urinary NAG levels in the chronic Pb-exposed group (Fig. 4a). Subgroup analyses, accounting for simultaneous exposure to other heavy metals and the country of reporting, yielded results consistent with the overall pooled findings, indicating that these variables did not entirely explain the observed heterogeneity across studies (Fig. S6). LOOMA demonstrated that excluding any individual study did not significantly influence the overall results, suggesting the robustness of the pooled observations (Fig. S7). Meta-regression analysis incorporating participant age as a covariate, did not substantially diminish heterogeneity or alter the outcomes. Furthermore, an asymmetric funnel plot suggests potential publication bias (p = 0.288), alongside other biases indicated by the contour-enhanced funnel plot (Fig. S8). Congruent with the pooled SMD, pooling urinary NAG values of the five studies reporting in mmol/hour/mmol creatinine revealed a difference of 1.66 (0.76–2.56, *I*^2^ = 97.21 %) units higher among Pb-exposed individuals (Fig. S9). Additionally, two studies [64], [37] reported higher urinary NAG (U/L) among Pb-exposed groups compared to controls.

### 4.4 Beta-2-Microglobulin (β_2_μG)

Seventeen [18] studies reported differences in β2μG levels among chronic Pb-exposed as compared to their respective comparator groups [39], [40], [44], [48], [50], [51], [52], [53], [54], [55], [57], [59], [61], [62], [63], [68], [74]. All but five studies [68], [61], [53], [59], [57] observed higher β_2_μG levels among chronic Pb-exposed individuals compared to controls, with some studies reporting statistically significant differences, consistent with the pooled estimate. Due to heterogeneous reporting of β_2_μG values (i.e. μg/g creatinine, μg/mmol creatinine and ng/mL using urinary sample and mg/L using serum sample), the SMD was calculated to estimate the pooled effect. A meta-analysis of the 17 revealed a pooled SMD of 1.5 (0.86–2.14, *I*^2^=98 %) higher β_2_μG levels in the chronic Pb-exposed group (Fig. 4b). Subgroup analyses, accounting for simultaneous exposure to other heavy metal(s) and the country of reporting, yielded results consistent with the overall pooled findings, indicating that these variables did not fully explain the observed heterogeneity across studies (Fig. S10). LOOMA demonstrated that excluding any individual study did not significantly influence the overall results, suggesting robustness in the findings (Fig. S11). Meta-regression analysis incorporating participant age as a covariate, did not substantially diminish heterogeneity or alter the outcomes. Furthermore, an asymmetric funnel plot suggests potential publication bias (*p* = 0.38) alongside other biases indicated by the contour-enhanced funnel plot (Fig. S12).

**Fig. 5:**
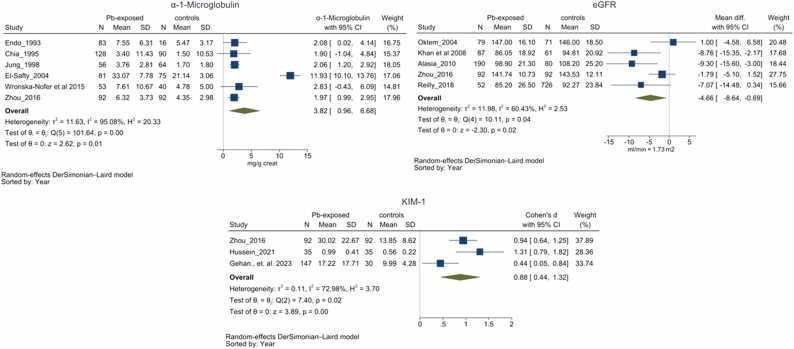
Forest plot for α-1-Microglobulin, effective glomerular filtration rate and Kidney Injury Molecule (KIM) – 1. Group differences in α-1-Microglobulin (5 A), effective glomerular filtration rate (5B) & Kidney Injury Marker - 1 (5 C) between Pb-exposed and unexposed group. Forest plot demonstrates significantly higher α-1-Microglobulin (3.82 mg/g creatinine with 95 % CI 0.96–6.68), KIM – 1 (0.88 SMD with 95 % CI 0.44 – 1.32) and significantly poor effective glomerular filtration rate (– 4.66 mL/min/1.73 m^2^ with 95 % CI −8.64–0.69) among Pb-exposed individuals, However the heterogeneity among the studies were unacceptably highly (*I*^2^ = 95.08 %, 72.98 % & 60.43 % respectively).

### 4.5 Alpha - 1 - Microglobulin (α1μG)

Six of the included studies provided data on urinary α1μG levels [60], [55], [52], [40], [39], [47]. All studies reported marginally higher α1μG in the Pb-exposed group compared to controls. The pooled mean difference indicated significantly higher α1μG levels among chronically Pb-exposed individuals, estimated at 3.82 mg/g creatinine (95 % CI 0.96–6.68 and *I*^2^=95.08 %) (Fig. 5a). Due to the limited number of studies available, sub group, meta-regression and funnel plot assessments were not performed.

### 4.6 Estimated Glomerular Filtration Rate (e-GFR)

Five of the included studies provided data on urinary e-GFR levels [42], [48], [38], [39]. All but Arslan Oktem et al. observed lower e-GFR among Pb-exposed individuals compared to controls [48]. The pooled mean difference observed was – 4.66 mL/min/1.73 m^2^ (-8.64–0.69 and *I*^2^ = 60.43 %) (Fig. 5b). Due to the limited number of studies available, sub group, meta-regression and funnel plot assessments were not performed.

### 4.7 Kidney Injury Molecule – 1 (KIM – 1)

Three of the included studies provided data on urinary KIM-1 levels [10], [33], [39]. All studies reported significantly higher KIM-1 in the exposed group compared to controls. Due to heterogeneous reporting of KIM-1 values (i.e. ng/g creatinine and ng/mL), the SMD was calculated to estimate the pooled effect. The SMD revealed significantly higher KIM-1 levels among chronically Pb-exposed individuals compared to the controls i.e. 0.88 (0.44–1.32 and *I*^2^=72.98 %) (Fig. 5c). Due to the limited number of studies available, sub group, meta-regression and funnel plot assessments were not performed.

### 4.8 Other markers of kidney function

Zhou et al. (2016) alone reported serum Cystatin - C, demonstrating significantly elevated levels (p = 0.001) of serum Cystatin - C in the Pb-exposed group compared to the control group [39]. Adejumo et al. was the only study to report NAGL levels between the two groups. The Pb-exposed group exhibited a trend of higher NAGL levels (*p* = 0.199) compared to the control [77]

## 5 Discussion

The systematic review explored the association between chronic Pb exposure and markers of kidney injury by analyzing primary studies that compared these markers between occupationally Pb-exposed individuals and control subjects without apparent Pb exposure. The results indicated significantly elevated levels of serum creatinine, NAG, α1μG, KIM-1, and β2μG in the Pb-exposed group compared to controls; however, notable heterogeneity was observed among the primary studies included in the review.

Blood lead levels were consistently higher in the Pb-exposed group across all studies, regardless of the duration or source of Pb exposure. Surprisngly, the control group, despite having no apparent Pb exposure, also exhibited detectable Pb levels in their blood, albeit significantly lower than those of the exposed group. Given that Pb has no recognized physiological role and its established toxicity (such as observed in current study), the presence of Pb in the blood of community residents without apparent exposure is a critical public health issue, underscoring prompt preventive interventions.

Pooled analysis revealed significantly higher urinary levels of kidney injury proteins (NAG, α_1_μG and β_2_μG) among chronically Pb-exposed workers compared to controls. Although pooled analyses weren’t permitted for Cystatin due to limited availability of primary studies, individual studies consistently reported significantly higher levels of this protein in Pb-exposed workers relative to controls. Current systematic review couldn’t identify studies reporting changes in ADMA, Podocalyxin, Nephrin, podocin and L-FABP, between the two groups. Notably, the Pb-exposed group exhibited significantly poorer pooled serum creatinine and eGFR compared to controls. However, in view of high heterogeneity among the included studies (*I*^2^ > 95 %), potential high publication biases (revealed by funnel plot and contour enhanced funnel plots) and the very low certainty of evidence, these findings mandates guarded interpretation. Furthermore, the limited availability of primary studies restricted the ability to perform meta-regression and subgroup analyses for some of the outcome parameters. These findings underscore the need for more robust and comprehensive studies to clarify the association between chronic Pb exposure and kidney injury markers.

Chronic Pb exposure results in its accumulation in solid organs such as bones, kidney, brain and liver. Since Pb is primarily excreted via urine, it’s posited that renal tissue experiences continuous insults as long as Pb remains in circulation. Preclinical, in-vitro and clinical studies have documented insults at various units / parts of nephrons due to chronic Pb exposure, manifesting as proximal tubular dysfunction, chronic interstitial nephritis and progressive chronic kidney disease ultimately culminating in end stage renal failure [39]. Urinary proteins such as KIM-1, β_2_μG, α_1_μG, Cystatin-C, ADMA, Podocalyxin, Nephrin, podocin, L-FABP, NAGL serve as sensitive and early markers of renal injury and / dysfunction, often detectable before deviations in serum creatinine and e-GFR [41]. Under the light of current observations, Pb-exposed workers experience proximal tubular injury (KIM-1, NAG) and dysfunction (β2μG, α1μG, Cystatin-C), prior to onset of obvious renal failure, as evidenced by e-GFR & serum creatinine levels not significantly differing from controls.

Recent evidence hints multiple mechanisms underlying the systemic toxicity induced by chronic Pb exposure, including it role as an endocrine disruptor, direct cellular toxicity and displacing divalent cations [2], [5], [78]. This study is perhaps the earliest to systematically review and demonstrate the nephrotoxic effects of chronic Pb exposure by examining its association with kidney injury markers Present results are to be cautiously interpreted with the background of limitations associated with systematically pooling observations from the primary studies viz. absence of high-powered studies, heterogeneity among them, non-uniform reporting and others. Further, present systematic review included studies of observational and cross sectional designs, with inherent high levels of risk of bias, fewer primary studies available for meta-regression, sub-group and sensitivity analysis and pooled mean differences with wide confidence intervals. These limitations pose significant challenges in interpretation our observations, including the potential for skewed pooled estimates due to publication bias, reduced robustness of results, and the inability to fully mitigate the high risk of bias inherent in cross sectional observational studies, ultimately impacting the overall quality of evidence. These limitations could be potentially addressed through future longitudinal studies that investigate markers of renal injury and dysfunction including those unavailable in the current review, among chronic lead exposed individuals. The longitudinal studies would provide deeper insights in terms of understanding dose – response relation between Pb exposure and early changes in kidney injury markers. They would also enable a deeper understanding of the kinetics and dynamics of the interaction between Pb exposure and these markers, while offering a clearer characterization of the initial renal insults. Such studies are essential for unravelling the underlying pathophysiology of Pb-induced nephrotoxicity. Given the potential nephrotoxicity, mandatory screening of Pb-exposed workers using sensitive renal injury markers is strongly recommended to facilitate early detection and intervention.

## 6 Conclusion

Current evidence suggests that chronic Pb exposure has nephrotoxic effects, with the proximal tubule being particularly susceptible. However, considering the limited availability of high-quality studies, further evidence from multicenter longitudinal studies incorporating additional investigations (markers of renal injury and dysfunction) is recommended to better understand the relation. Present observations underscore the importance of policymakers formulating regulations that mandate periodic screening for early markers of renal injury and / dysfunction besides conventional kidney function markers among individuals with chronic Pb exposure.

## Ethics approval

Not applicable, the study was conceived from secondary data available from various literature sources.

## Funding

The study did not receive any funding / financial support for this work. The authors declare that no funds, grants, or other support were received during the preparation of this manuscript.

## CRediT authorship contribution statement

**Ankit Viramgami:** Writing – review & editing, Methodology, Investigation, Data curation, Conceptualization. **Bhavani Shankara Bagepally:** Writing – review & editing, Supervision, Software, Methodology, Formal analysis, Conceptualization. **Kuldip Upadhyay:** Writing – review & editing, Writing – original draft, Methodology, Investigation, Data curation, Conceptualization. **Rakesh Balachandar:** Writing – original draft, Supervision, Methodology, Investigation, Formal analysis, Data curation, Conceptualization.

## Declaration of Competing Interest

The authors declare that they have no known competing financial interests or personal relationships that could have appeared to influence the work reported in this paper.

## Data Availability

Data will be made available on request.

## Footnotes

Appendix A Supplementary data associated with this article can be found in the online version at doi:10.1016/j.toxrep.2024.101837.
