# Supplementary material for: Association between chronic lead exposure and markers of kidney injury: A systematic review and meta-analysis
Source: Toxicol Rep. 2024 Nov 29;13:101837. doi: 10.1016/j.toxrep.2024.101837 (PMC11664089; doi:10.1016/j.toxrep.2024.101837)
Supplement: Supplementary file 1 — Supplementary material [file mmc1.docx]

Supplementary materials

Contents

[Supplement figure 1: Subgroup analysis for BLL 2](#_Toc161837662)

[Supplement figure 2: Funnel plot of BLL 3](#_Toc161837663)

[Supplement figure 3: Subgroup analysis for creatinine 4](#_Toc161837664)

[Supplement figure 4: Leave-One-Out-Analysis for creatinine 5](#_Toc161837665)

[Supplement figure 5: Funnel plot for creatinine 6](#_Toc161837666)

[Supplement figure 6: Subgroup analysis for NAG (SMD) 7](#_Toc161837667)

[Supplement figure 7: Leave-One-Out-Analysis for NAG (SMD) 8](#_Toc161837668)

[Supplement figure 8: Funnel plot for NAG (SMD) 9](#_Toc161837669)

[Supplement Figure 9: Forest plot NAG (mmol/hour/mmol of creatinine) 10](#_Toc161837670)

[Supplement figure 10: Subgroup analysis for Beta-2-Microglobulin (SMD) 11](#_Toc161837671)

[Supplement figure 11: Leave-One-Out-Analysis for Beta-2-Microglobulin (SMD) 12](#_Toc161837672)

[Supplement figure 12: Funnel plot for Beta-2-Microglobulin (SMD) 13](#_Toc161837673)

[Supplementary Table 1a: Search strategy at pubmed medline digital library 14](#_Toc161837674)

[Supplementary table 1b: Search strategy at Embase digital library 15](#_Toc161837675)

[Supplementary table 1c: Search strategy at Scopus digital library 17](#_Toc161837676)

[Supplementary table 2: Summary of the pooled estimates of outcome parameters 18](#_Toc161837677)

[Supplementary table 3: Summary of findings of GRADE assessment 19](#_Toc161837678)

[SOP for risk of bias assessment using the Newcastle-Ottawa quality assessment scale 20](#_Toc161837679)

## Supplement figure 1: Subgroup analysis for BLL


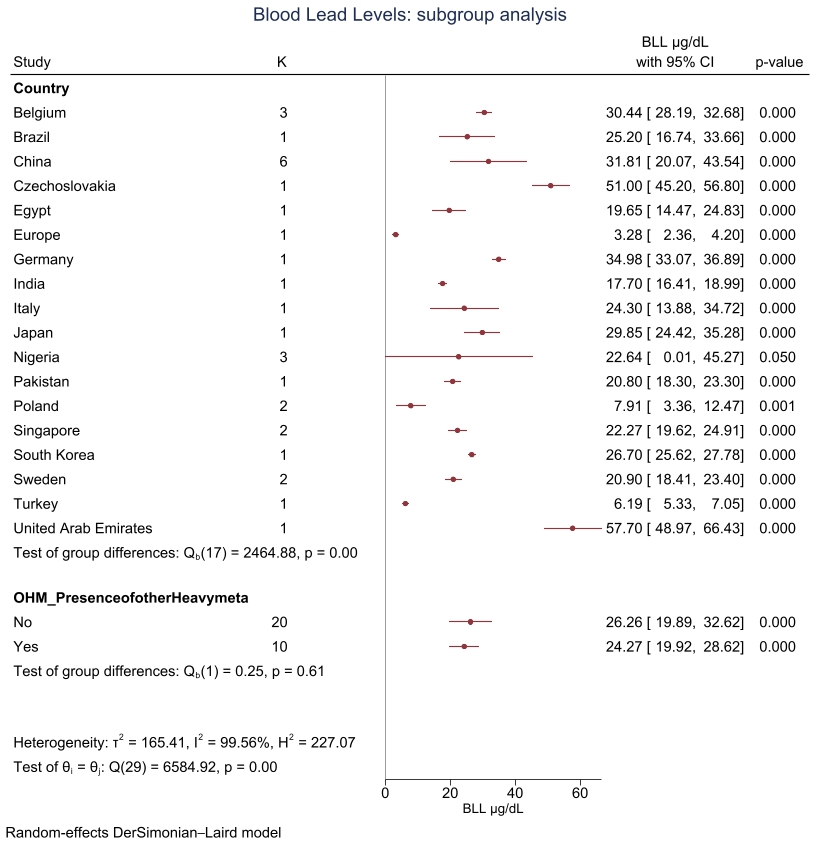


(Legends / footnotes): Group difference in BLL between the occupationally Pb exposed and unexposed workers. The sub group analysis based on country of reporting, and simultaneous exposure to other heavy metals at workplaces.

## Supplement figure 2: Funnel plot of BLL


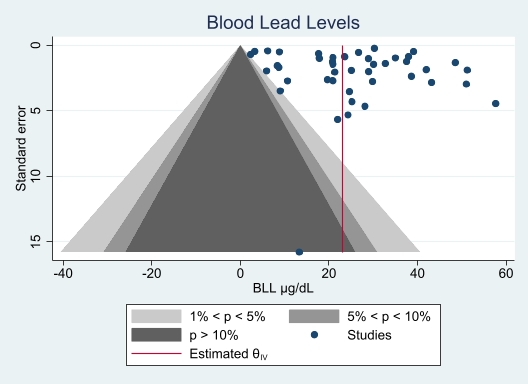

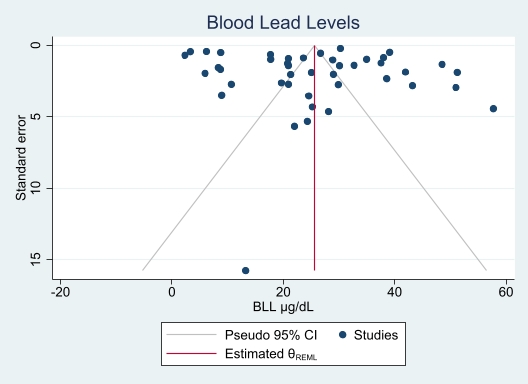


(Legend / footnote) X axis represents the estimated mean difference in BLL between the 2 groups and y axis represents the precision of the measure (standard error). The (two-sided) statistical significance of any point on a funnel plot can be calculated by considering the estimated measure and precision. Various shades in the Contour - enhanced funnel plot (right image) represent the levels of statistical significance (from inside -out > 10%, 1- 5 % &<1%).

## Supplement figure 3: Subgroup analysis for creatinine


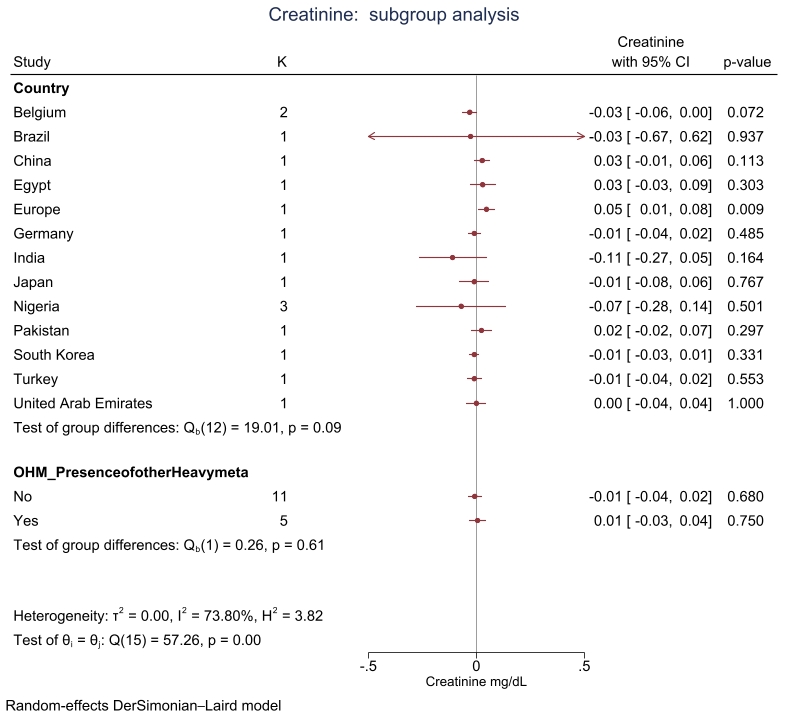


(Legends / footnotes): Group difference in creatinine between the occupationally Pb exposed and unexposed workers. The sub group analysis based on country of reporting, and simultaneous exposure to other heavy metals at workplaces.

## Supplement figure 4: Leave-One-Out-Analysis for creatinine


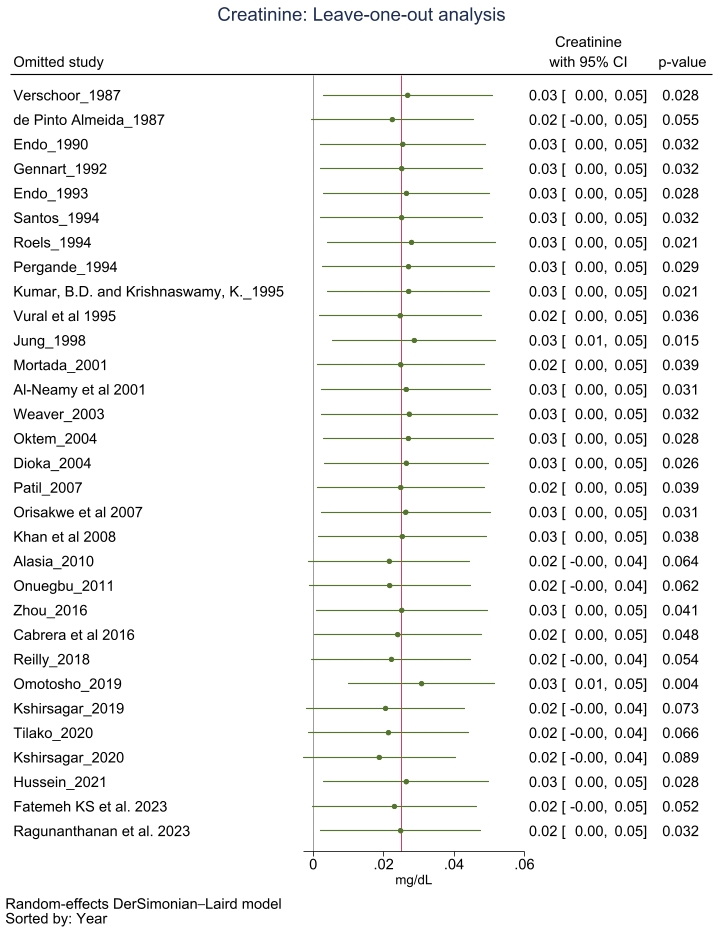


Group difference in creatinine between the occupationally Pb exposed and unexposed workers.

##
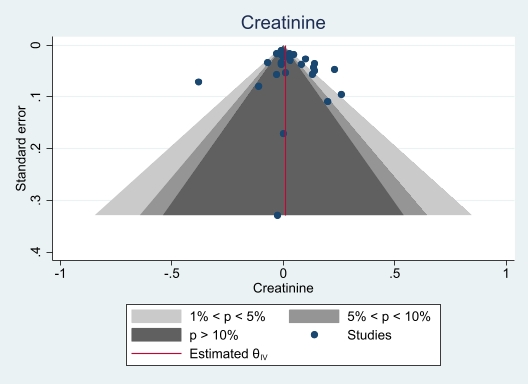
Supplement figure 5: Funnel plot for creatinine


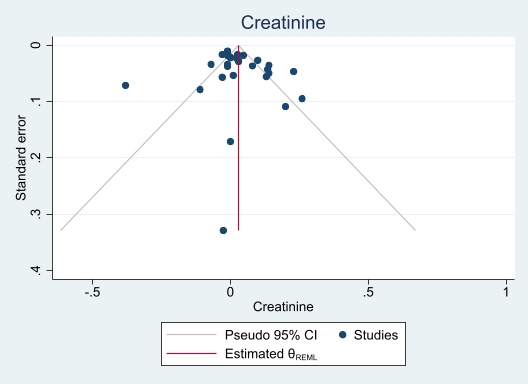


(Legend / footnote) X axis represents the estimated mean difference in serum creatinine between the 2 groups and y axis represents the precision of the measure (standard error). The (two-sided) statistical significance of any point on a funnel plot can be calculated by considering the estimated measure and precision. Various shades in the Contour - enhanced funnel plot (right image) represent the levels of statistical significance (from inside -out > 10%, 1- 5 % &<1%).

## Supplement figure 6: Subgroup analysis for NAG (SMD)


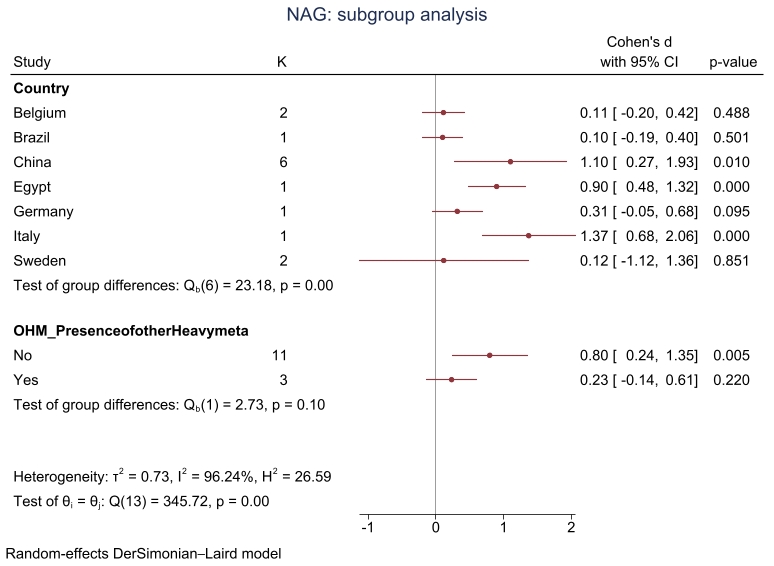


(Legends / footnotes): Group difference in NAG (reported as standardized mean differences) between the occupationally Pb exposed and unexposed workers. The sub group analysis based on country of reporting, and simultaneous exposure to other heavy metals at workplaces.

## Supplement figure 7: Leave-One-Out-Analysis for NAG (SMD)


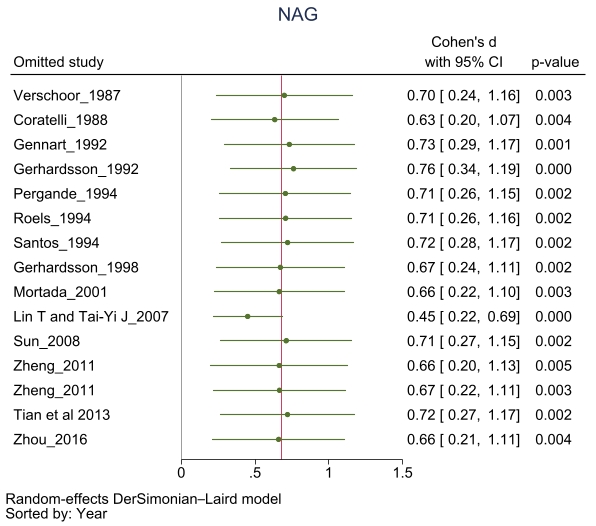


Group difference in NAG (reported as standardized mean differences) between the occupationally Pb exposed and unexposed workers.

## Supplement figure 8: Funnel plot for NAG (SMD)


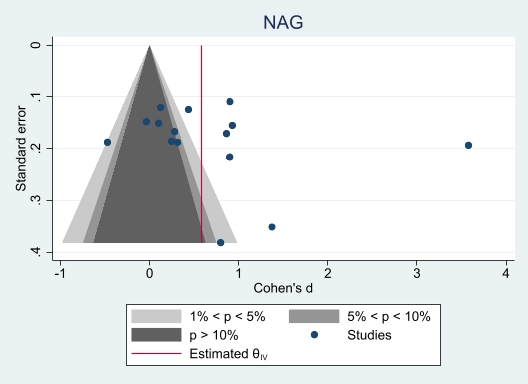

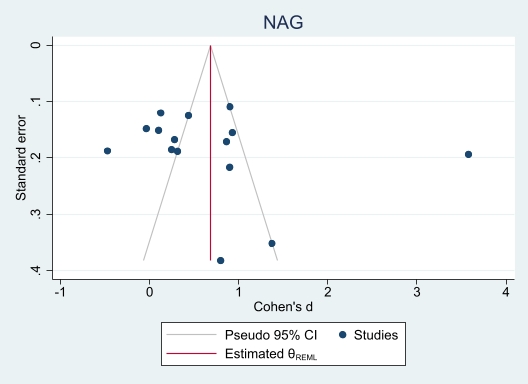


(Legend / footnote) X axis represents the estimated mean difference in NAG (estimated as standardized mean difference) between the 2 groups and y axis represents the precision of the measure (standard error). The (two-sided) statistical significance of any point on a funnel plot can be calculated by considering the estimated measure and precision. Various shades in the Contour - enhanced funnel plot (right image) represent the levels of statistical significance (from inside -out > 10%, 1- 5 % &<1%).

## Supplement Figure 9: Forest plot NAG (mmol/hour/mmol of creatinine)


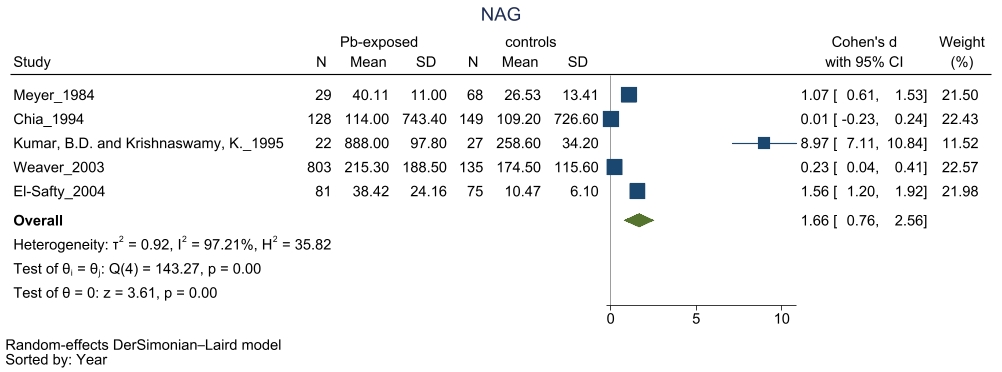


(Legends / footnotes) Group difference in the urinary NAG values (reported as (mmol/hour/mmol of creatinine) among Pb exposed and unexposed workers.

## Supplement figure 10: Subgroup analysis for Beta-2-Microglobulin (SMD)


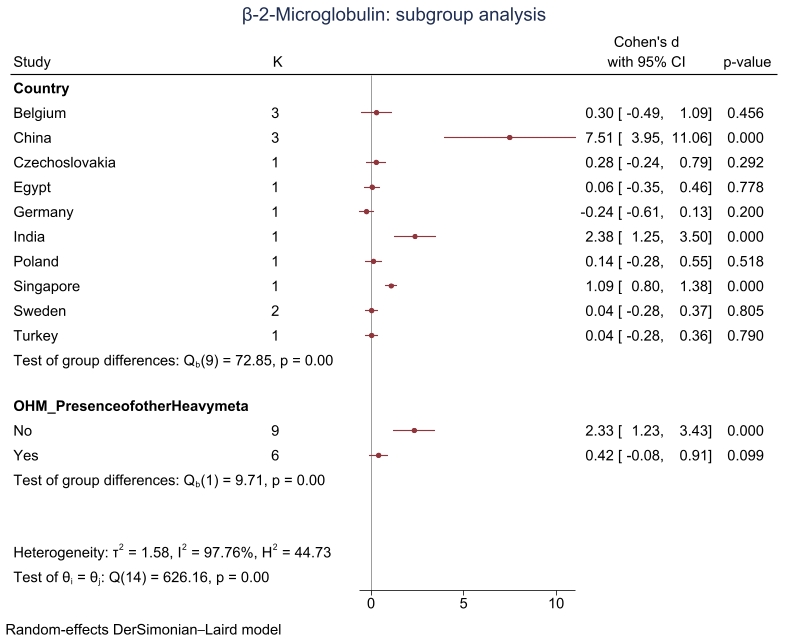


(Legends / footnotes): Group difference in Beta-2-Microglobulin (reported as standardized mean differences) between the occupationally Pb exposed and unexposed workers. The sub group analysis based on country of reporting, and simultaneous exposure to other heavy metals at workplaces.

## Supplement figure 11: Leave-One-Out-Analysis for Beta-2-Microglobulin (SMD)


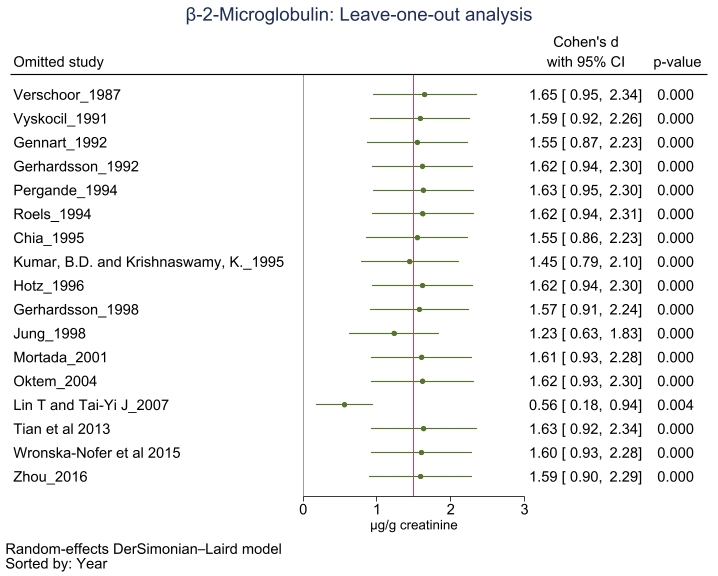


Group difference in Beta-2-Microglobulin (reported as standardized mean differences) between the occupationally Pb exposed and unexposed workers.

## Supplement figure 12: Funnel plot for Beta-2-Microglobulin (SMD)


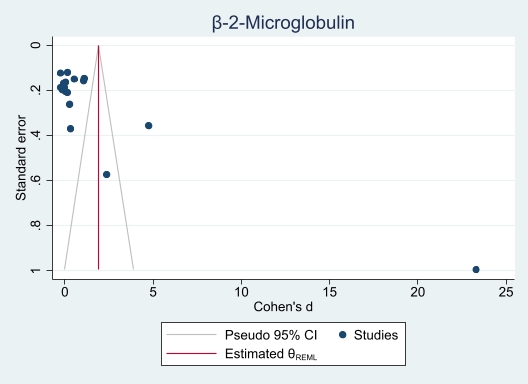

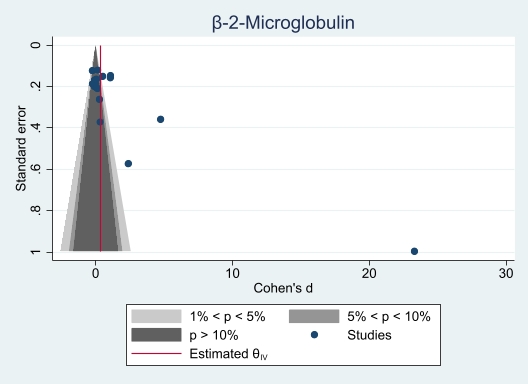


(Legend / footnote) X axis represents the estimated mean difference in Beta-2-Microglobulin (pooled as standardized mean difference) between the 2 groups and y axis represents the precision of the measure (standard error). The (two-sided) statistical significance of any point on a funnel plot can be calculated by considering the estimated measure and precision. Various shades in the Contour - enhanced funnel plot (right image) represent the levels of statistical significance (from inside -out > 10%, 1- 5 % &<1%).

## Supplementary Table 1a: Search strategy at pubmed medline digital library

| **PICOS** | **Pubmed-Medline** |
| --- | --- |
| 1. Lead | "Lead"[Mesh] OR (lead acetate) OR (lead tetraoxide) OR (lead tetraacetate) OR (lead chromate) OR (lead phosphate) OR (lead oxide) OR (lead silicate) OR (tetraethyl lead) OR (lead poisoning) OR “plumbum” OR (tetraethyl lead) |
| 2. Chronic kidney function indicators | rena* OR kidne* OR “CKD”[Title/Abstract] OR “CKF”[Title/Abstract] OR “CRD”[Title/Abstract] OR “CRF”[Title/Abstract] OR “ESKD”[Title/Abstract] OR dialysis OR creatini* OR (Cystatin C) OR (beta-Trace protein) OR (glomeru* filtration rate) OR “gfr”[Title/Abstract] OR (glomerulofiltration rate) OR (neutrophil gelatinase associated lipocalin) OR “NGAL”[Title/Abstract] OR (kidney injury molecule-1) OR KIM-1 OR “N-acetyl-beta-O-glucosaminidase"[All Fields] OR "NAG"[Title/Abstract] OR L-FABP OR (liver-type fatty acid-binding protein) OR Podocin OR Nephrin OR Podocalyxin OR ADMA OR (asymmetric dimethylarginine) |
|  | 1 & 2 |
|  | 1 & 2 Filters: Humans |

## Supplementary table 1b: Search strategy at Embase digital library

| **PICOS** | **Embase** |
| --- | --- |
| Lead | 'lead'/exp OR 'plumbum' OR 'radioisotope, lead' |
| Chronic kidney function indicators | 'kidney disease'/exp OR 'disease, kidney' OR 'kidney disease' OR 'kidney diseases' OR 'kidney disorder' OR 'kidney pathology' OR 'nephropathy' OR 'perinephritis' OR 'perirenal infection' OR 'renal disease' OR 'renal disorder' OR 'unilateral kidney disease' OR 'chronic kidney failure'/exp OR 'chronic kidney disease' OR 'chronic kidney disorder' OR 'chronic kidney failure' OR 'chronic kidney insufficiency' OR 'chronic nephropathy' OR 'chronic renal disease' OR 'chronic renal failure' OR 'chronic renal insufficiency' OR 'kidney chronic failure' OR 'kidney disease, chronic' OR 'kidney failure, chronic' OR 'kidney function, chronic disease' OR 'renal insufficiency, chronic' OR 'dialysis'/exp OR 'acute dialysis' OR 'coil dialysis' OR 'dialysis' OR 'flow dialysis' OR 'creatinine'/exp OR '1 methylglycocyamidine' OR '1 methylhydantoin 1 imide' OR '2 imino 1 methyl 4 imidazolinone' OR 'creatinin' OR 'creatinine' OR 'creatinine hydrochloride' OR 'kreatinine' OR 'methylglycocyamimine' OR 'cystatin c'/exp OR 'cystatin 3' OR 'cystatin c' OR 'gamma trace' OR 'neuroendocrine basic polypeptide' OR 'post gamma globulin' OR 'beta trace protein'/exp OR 'glomerulus filtration rate'/exp OR 'gfr (glomerulus filtration rate)' OR 'glomerular filtration rate' OR 'glomerulofiltration rate' OR 'glomerulus filtration rate' OR 'kidney gfr' OR 'kidney glomerulus filtration rate' OR 'neutrophil gelatinase associated lipocalin'/exp OR '24p3 protein' OR 'lcn2 protein' OR 'ngal' OR 'ngal protein' OR 'sip24' OR 'sip24 protein' OR 'lipocalin 2' OR 'lipocalin 24p3' OR 'lipocalin-2' OR 'neutrophil gelatinase associated lipocalin' OR 'protein 24p3' OR 'protein lcn2' OR 'protein ngal' OR 'protein sip24' OR 'siderochalin' OR 'superinducible protein 24' OR 'uterocalin' OR 'kidney injury molecule 1 protein'/exp OR 'n acetyl beta glucosaminidase'/exp OR 'acetyl beta d glucosaminase' OR 'beta acetyl glucosaminidase' OR 'beta acetylglucosaminidase' OR 'beta n acetyl d glucosaminidase' OR 'beta n acetyl dextro glucosaminidase' OR 'beta n acetyl glucosaminidase' OR 'beta n acetylglucosaminidase' OR 'di n acetylchitobiase' OR 'e.c. 3.2.1.30' OR 'e.c. 3.2.1.96' OR 'endo beta n acetylglucosaminidase' OR 'endoglycosidase f' OR 'mannosyl glycoprotein endo beta n acetylglucosaminidase' OR 'mannosyl-glycoprotein endo-beta-n-acetylglucosaminidase' OR 'n acetyl beta d glucosaminidase' OR 'n acetyl beta d glucosaminide n acetylglucosaminohydrolase' OR 'n acetyl beta dextro glucosaminidase' OR 'n acetyl beta glucosaminidase' OR 'n beta acetylglucosaminidase' OR 'nag' OR 'l fabp' OR 'liver type fatty acid binding protein'/exp OR 'podocin'/exp OR 'nphs2 protein' OR 'podocin' OR 'protein nphs2' OR 'nephrin'/exp OR 'nphs1 protein' OR 'nephrin' OR 'protein nphs1' OR 'podocalyxin'/exp OR 'n(g),n(g) dimethylarginine'/exp OR '6 n, n dimethylarginine' OR 'adma' OR 'asymmetric dimethylarginine' OR 'asymmetrical dimethylarginine' OR 'n (g), n (g) dimethyl levo arginine' OR 'n 6, n 6 dimethylarginine' OR 'n(g),n(g) dimethylarginine' OR 'ng, ng dimethylarginine' |
|  | 1 & 2 |
|  | #3 AND ('article'/it OR 'article in press'/it) |

## Supplementary table 1c: Search strategy at Scopus digital library

| **PICOS** | **SCOPUS** |
| --- | --- |
| Lead | TITLE-ABS-KEY ( "lead acetate" OR "lead tetraoxide" OR "lead tetraacetate" OR "lead chromate" OR "lead phosphate" OR "lead oxide" OR "lead silicate" OR "tetraethyl lead" OR "lead poisoning" OR "Pb" OR "plumbum" OR "tetraethyl" ) |
| Chronic kidney function indicators | TITLE-ABS-KEY ( rena* OR kidne* OR "CKD" OR "CKF" OR "CRD" OR "CRF" OR "ESKD" OR dialysis OR creatini* OR ( cystatin AND c ) OR ( beta-trace AND protein ) OR ( glomeru* AND filtration AND rate ) OR "gfr" OR ( glomerulofiltration AND rate ) OR ( neutrophil AND gelatinase AND associated AND lipocalin ) OR "NGAL" OR ( kidney AND injury AND molecule-1 ) OR kim-1 OR "N-acetyl-beta-O-glucosaminidase" OR "NAG" OR l-fabp OR ( liver-type AND fatty AND acid-binding AND protein ) OR podocin OR nephrin OR podocalyxin OR adma OR ( asymmetric AND dimethylarginine ) ) |
|  | 1 & 2 |
|  | ( TITLE-ABS-KEY ( "lead acetate" OR "lead tetraoxide" OR "lead tetraacetate" OR "lead chromate" OR "lead phosphate" OR "lead oxide" OR "lead silicate" OR "tetraethyl lead" OR "lead poisoning" OR "Pb" OR "plumbum" OR "tetraethyl" ) ) AND ( TITLE-ABS-KEY ( rena* OR kidne* OR "CKD" OR "CKF" OR "CRD" OR "CRF" OR "ESKD" OR dialysis OR creatini* OR ( cystatin AND c ) OR ( beta-trace AND protein ) OR ( glomeru* AND filtration AND rate ) OR "gfr" OR ( glomerulofiltration AND rate ) OR ( neutrophil AND gelatinase AND associated AND lipocalin ) OR "NGAL" OR ( kidney AND injury AND molecule-1 ) OR kim-1 OR "N-acetyl-beta-O-glucosaminidase" OR "NAG" OR l-fabp OR ( liver-type AND fatty AND acid-binding AND protein ) OR podocin OR nephrin OR podocalyxin OR adma OR ( asymmetric AND dimethylarginine ) ) ) AND ( LIMIT-TO ( DOCTYPE , "ar" ) ) AND ( LIMIT-TO ( EXACTKEYWORD , "Article" ) OR LIMIT-TO ( EXACTKEYWORD , "Human" ) ) AND ( LIMIT-TO ( SRCTYPE , "j" ) ) AND ( EXCLUDE ( EXACTKEYWORD , "Nonhuman" ) ) |

## Supplementary table 2: Summary of the pooled estimates of outcome parameters

| S.N | Outcome parameters | No. studies | Units / analysis | Mean difference (95% CI) | Heterogeneity (*I^2^* %) |
| --- | --- | --- | --- | --- | --- |
|  | Creatinine | 31 | mg/dL | 0.03 (0.00 to 0.05) | 78.72 |
|  | NAG | 14 | U/ g creatinine | 3.03 (1.52 to 4.54) | 98.39 |
|  | NAG | 5 | mmol/Hr/mmol creatinine | 1.66 (0.76 to 2.56) | 97.21 |
|  | NAG | 4 | U / mmol creatinine | 0.19 (-0.06 to 0.44) | 90.2 |
|  | NAG | 15 | SMD | 0.68 (0.26 to 1.1) | 95.97 |
|  | α-1-Microglobulin | 6 | mg / g creatinine | 3.82 (0.96 to 6.68) | 95.08 |
|  | β-2-Microglobulin | 2 | µg / liter | -0.3 (-0.61 to 0.01) | 0.00 |
|  | β-2-Microglobulin | 3 | µg / mmol creatinine | -0.6 (-4.32 to 3.12) | 11.27 |
|  | β-2-Microglobulin | 12 | µg / g creatinine | 18.88 (1.14 to 36.29) | 99.92 |
|  | β-2-Microglobulin | 14 | SMD | 1.5 (0.86 to 2.14) | 98 |
|  | e-GFR | 5 | ml/min/1.73 m^2^ | -4.66 (-8.64 to 0.69) | 60.43 |
|  | Cystatin - C | 1 | Mg/L urine | *Pb > C ( *p* = 0.001) | - |
|  | KIM-1 | 3 | ng/g creat | 0.88 (0.44 to 1.32) | 72.98 |
|  | NAGL | 1 | Ng/mL | *Pb > C ( *p* = 0.199) | - |
|  | ADMA, Podocalyxin, Nephrin, podocin, L-FABP, | 0 | - | Not available | - |

*Pb exposed group exhibited relatively higher levels as compared to the control group

## Supplementary table 3: Summary of findings of GRADE assessment

Evidence Profile using Grading of Recommendation, Assessment, Development, and Evaluation (GRADE) instrument

**Population:** All adults

**Exposure (intervention):** Chronic lead (Pb) exposure by virtue of occupation

**Control:** No obvious exposure to Pb

**Outcomes:** Biomarkers of chronic kidney disease.

| **Outcome: biomarkers of chronic kidney disease (assessed with mean differences in the levels using meta-analysis)** | | | | | | | | | |
| --- | --- | --- | --- | --- | --- | --- | --- | --- | --- |
| **Quality assessment** | | | | | | **Summary of findings** | | | **Comments** |
| **No of studies** | **Risk of Bias** | **Inconsistency** | **Indirectness** | **Imprecision** | **Publication Bias** | **Mean difference** | | **Certainty/Quality** |  |
|  |  |  |  |  |  | **INB** | **95%CI** |  |  |
| Serum creatinine levels among chronic Pb exposed as compared to those without obvious Pb exposure (assessed with meta-analysis). | | | | | | | | | |
| 31 | serious | Serious ^a^ | serious | Serious ^b^ | likely | 0.03 mg/dL | 0.0 to 0.05 | ⊕🌕🌕🌕  Very Low |  |
| Urinary N-Acetyl-beta-D-Glucosaminidase (NAG) levels among chronic Pb exposed as compared to those without obvious Pb exposure (assessed with meta-analysis). | | | | | | | | | |
| 15 | serious | Serious ^a^ | serious | Serious ^b^ | likely | 0.68* | 0.26 to 1.1 | ⊕🌕🌕🌕  Very Low |  |
| Urinary Beta-2-Microglobulin levels among chronic Pb exposed as compared to those without obvious Pb exposure (assessed with meta-analysis). | | | | | | | | | |
| 17 | serious | Serious ^a^ | serious | Serious ^b^ | likely | 1.5* | 0.86 to 2.14 | ⊕🌕🌕🌕  Very Low |  |
| Urinary Alpha - 1 - Microglobulin levels among chronic Pb exposed as compared to those without obvious Pb exposure (assessed with meta-analysis). | | | | | | | | | |
| 6 | serious | Serious ^a^ | serious | Serious ^b^ | likely | 3.82 mg/g creatinine | 0.96 to 6.68 | ⊕🌕🌕🌕  Very Low |  |
| Estimated glomerular filtration rate among chronic Pb exposed as compared to those without obvious Pb exposure (assessed with meta-analysis). | | | | | | | | | |
| 5 | serious | Serious ^a^ | serious | Serious ^b^ | likely | – 4.66 ml/min/1.73 m2 | -8.64 to 0.69) | ⊕🌕🌕🌕  Very Low |  |
| Urinary Kidney Injury Molecule-1 levels among chronic Pb exposed as compared to those without obvious Pb exposure (assessed with meta-analysis) | | | | | | | | | |
| 3 | serious | Serious ^a^ | serious | Serious ^b^ | likely | 0.88ng/mL | 0.44 – 1.32 | ⊕🌕🌕🌕  Very Low |  |

^a^ high heterogeneity *I*^2^ > 90% ^b^ studies included have reported wide confidence intervals *standardized mean difference

**GRADE Working Group grades of evidence**
**High certainty:** we are very confident that the true effect lies close to that of the estimate of the effect.
**Moderate certainty:** we are moderately confident in the effect estimate; the true effect is likely to be close to the estimate of the effect, but there is a possibility that it is substantially different.
**Low certainty:** our confidence in the effect estimate is limited; the true effect may be substantially different from the estimate of the effect.
**Very low certainty:** we have very little confidence in the effect estimate; the true effect is likely to be substantially different from the estimate of effect

## SOP for risk of bias assessment using the Newcastle-Ottawa quality assessment scale

Note: A study can be awarded a maximum of one star for each numbered item within the Selection and Exposure categories. A maximum of two stars can be given for Comparability.

**Selection**

1) Is the case definition adequate?

a) Yes, with independent validation *****:

- Participants recruited at their workplace known to have Pb exposure
- Confirmed their employment with employer’s documents (eg ID card, Appointment Letter, etc.)

b) Yes, eg record linkage or based on self-reports:

- Participants were recruited from sites other than their workplace exposure, based on history and employer’s documents (eg ID card, Appointment Letter, etc.)

c) No description: The study has no description of the above mentioned content

2) Representativeness of the cases

a) Consecutive or obviously representative series of cases *****

- Participants with documented evidence of occupationally Pb exposure, demonstration of Pb exposure at the workplace, by reporting the ambient Pb levels at the workplace
- No past history of renal dysfunction

b) Potential for selection biases or not stated

- Participants with co-exposure to other heavy metals may alter their renal function.
- Patients with pre-existing Pb toxicity
- Participants with pre-existing renal dysfunction
- No description of the above parameters in the study

3) Selection of Controls

a) Community controls *****

- Apparently healthy, without a previous history of occupational Pb exposure and renal dysfunction.
- Demographically match with case participants in age, sex and socio-economic status.

b) Hospital controls

- Participants recruited from the same organization or other organization involving processing Pb, however the participant’s work profile does not have direct exposure to Pb.

c) No description

4) Definition of Controls

a) No history of disease (endpoint) *****

- Occupationally non-exposed to Pb
- No history of previous Pb exposure/retired from job with pb exposure
- No history of renal dysfunction manifestation

b) No description of source

**Comparability**

1) Comparability of cases and controls on the basis of the design or analysis

a) Study controls for blood Pb levels, heavy metals, occupational factors, renal related symptoms*****

b) Study controls for any additional factor such as co-morbidities, substance use patterns, socio-economic patterns ***** (This criterion could be modified to indicate specific control for a second important factor)

**Exposure**

1) Ascertainment of exposure

a) Secure record *****

- Assessment of BLL, ambient Pb levels, heavy metals, heavy metals in the blood

b) Structured interview where blind to case/control status *****

- Whether participants being assessed for the current study were blinded to their exposure status or the outcome parameters being assessed

c) Interview not blinded to case/control status

- Whether participants being assessed for the current study were blinded to their exposure status or the outcome parameters being assessed during the interview (like the type of job, product or process description, working department, duration, etc.)

d) written self-report or medical record only

e) No description

Same method of ascertainment for cases and controls: Whether the tools / investigations / parameters used to classify the participants as exposed were used for categorizing the control participants as well

a) Yes *****

b) No

2) Non-Response rate:

Whether the missing data / non-response data were present in the same fraction / handled the in similar fashion among the exposed and the control group,

a) Same rate for both groups *****

b) Non-respondents described

c) Rate different and no designation

| **Section and Topic** | **Item #** | **Checklist item** | **Location where item is reported** |
| --- | --- | --- | --- |
| **TITLE** | | |  |
| Title | 1 | Identify the report as a systematic review. | 1 |
| **ABSTRACT** | | |  |
| Abstract | 2 | See the PRISMA 2020 for Abstracts checklist. | 3 |
| **INTRODUCTION** | | |  |
| Rationale | 3 | Describe the rationale for the review in the context of existing knowledge. | 5 |
| Objectives | 4 | Provide an explicit statement of the objective(s) or question(s) the review addresses. | 5 |
| **METHODS** | | |  |
| Eligibility criteria | 5 | Specify the inclusion and exclusion criteria for the review and how studies were grouped for the syntheses. | 6 |
| Information sources | 6 | Specify all databases, registers, websites, organisations, reference lists and other sources searched or consulted to identify studies. Specify the date when each source was last searched or consulted. | 6 |
| Search strategy | 7 | Present the full search strategies for all databases, registers and websites, including any filters and limits used. | Sup table 1 |
| Selection process | 8 | Specify the methods used to decide whether a study met the inclusion criteria of the review, including how many reviewers screened each record and each report retrieved, whether they worked independently, and if applicable, details of automation tools used in the process. | 6, 7 |
| Data collection process | 9 | Specify the methods used to collect data from reports, including how many reviewers collected data from each report, whether they worked independently, any processes for obtaining or confirming data from study investigators, and if applicable, details of automation tools used in the process. | 7 |
| Data items | 10a | List and define all outcomes for which data were sought. Specify whether all results that were compatible with each outcome domain in each study were sought (e.g. for all measures, time points, analyses), and if not, the methods used to decide which results to collect. | 7, 8 |
|  | 10b | List and define all other variables for which data were sought (e.g. participant and intervention characteristics, funding sources). Describe any assumptions made about any missing or unclear information. | 7 |
| Study risk of bias assessment | 11 | Specify the methods used to assess risk of bias in the included studies, including details of the tool(s) used, how many reviewers assessed each study and whether they worked independently, and if applicable, details of automation tools used in the process. | 8, 9 |
| Effect measures | 12 | Specify for each outcome the effect measure(s) (e.g. risk ratio, mean difference) used in the synthesis or presentation of results. | Sup table 2 |
| Synthesis methods | 13a | Describe the processes used to decide which studies were eligible for each synthesis (e.g. tabulating the study intervention characteristics and comparing against the planned groups for each synthesis (item #5)). | 8 |
|  | 13b | Describe any methods required to prepare the data for presentation or synthesis, such as handling of missing summary statistics, or data conversions. | 8 |
|  | 13c | Describe any methods used to tabulate or visually display results of individual studies and syntheses. | 8 |
|  | 13d | Describe any methods used to synthesize results and provide a rationale for the choice(s). If meta-analysis was performed, describe the model(s), method(s) to identify the presence and extent of statistical heterogeneity, and software package(s) used. | 8 |
|  | 13e | Describe any methods used to explore possible causes of heterogeneity among study results (e.g. subgroup analysis, meta-regression). | 8 |
|  | 13f | Describe any sensitivity analyses conducted to assess robustness of the synthesized results. | 8 |
| Reporting bias assessment | 14 | Describe any methods used to assess risk of bias due to missing results in a synthesis (arising from reporting biases). | 8. 9 |
| Certainty assessment | 15 | Describe any methods used to assess certainty (or confidence) in the body of evidence for an outcome. | 9 |
| **RESULTS** | | |  |
| Study selection | 16a | Describe the results of the search and selection process, from the number of records identified in the search to the number of studies included in the review, ideally using a flow diagram. | 9 |
|  | 16b | Cite studies that might appear to meet the inclusion criteria, but which were excluded, and explain why they were excluded. | 9 |
| Study characteristics | 17 | Cite each included study and present its characteristics. | 10 |
| Risk of bias in studies | 18 | Present assessments of risk of bias for each included study. | 10 |
| Results of individual studies | 19 | For all outcomes, present, for each study: (a) summary statistics for each group (where appropriate) and (b) an effect estimate and its precision (e.g. confidence/credible interval), ideally using structured tables or plots. | 11 - 14 |
| Results of syntheses | 20a | For each synthesis, briefly summarise the characteristics and risk of bias among contributing studies. | 10 |
|  | 20b | Present results of all statistical syntheses conducted. If meta-analysis was done, present for each the summary estimate and its precision (e.g. confidence/credible interval) and measures of statistical heterogeneity. If comparing groups, describe the direction of the effect. | 11 - 14 |
|  | 20c | Present results of all investigations of possible causes of heterogeneity among study results. | 11 – 14 |
|  | 20d | Present results of all sensitivity analyses conducted to assess the robustness of the synthesized results. | 11 – 14 |
| Reporting biases | 21 | Present assessments of risk of bias due to missing results (arising from reporting biases) for each synthesis assessed. | 10 |
| Certainty of evidence | 22 | Present assessments of certainty (or confidence) in the body of evidence for each outcome assessed. | 11 |
| **DISCUSSION** | | |  |
| Discussion | 23a | Provide a general interpretation of the results in the context of other evidence. | 15 – 16 |
|  | 23b | Discuss any limitations of the evidence included in the review. | 16 |
|  | 23c | Discuss any limitations of the review processes used. | 15 |
|  | 23d | Discuss implications of the results for practice, policy, and future research. | 16 |
| **OTHER INFORMATION** | | |  |
| Registration and protocol | 24a | Provide registration information for the review, including register name and registration number, or state that the review was not registered. | 6 |
|  | 24b | Indicate where the review protocol can be accessed, or state that a protocol was not prepared. | 6 |
|  | 24c | Describe and explain any amendments to information provided at registration or in the protocol. | 6 |
| Support | 25 | Describe sources of financial or non-financial support for the review, and the role of the funders or sponsors in the review. | 18 |
| Competing interests | 26 | Declare any competing interests of review authors. | 18 |
| Availability of data, code and other materials | 27 | Report which of the following are publicly available and where they can be found: template data collection forms; data extracted from included studies; data used for all analyses; analytic code; any other materials used in the review. | 18 |

*From:*  Page MJ, McKenzie JE, Bossuyt PM, Boutron I, Hoffmann TC, Mulrow CD, et al. The PRISMA 2020 statement: an updated guideline for reporting systematic reviews. BMJ 2021;372:n71. doi: 10.1136/bmj.n71

For more information, visit: <http://www.prisma-statement.org/>
